# Seminal CD38 Enhances Human Sperm Capacitation through Its Interaction with CD31

**DOI:** 10.1371/journal.pone.0139110

**Published:** 2015-09-25

**Authors:** Byung-Ju Kim, Dae-Ryoung Park, Tae-Sik Nam, Seo Ho Lee, Uh-Hyun Kim

**Affiliations:** 1 National Creative Research Laboratory for Ca2+ Signaling Network, Chonbuk National University Medical School, Jeonju, 561–180, Korea; 2 Department of Biochemistry, Chonbuk National University Medical School, Jeonju, Korea; 3 Institute of Cardiovascular Research, Chonbuk National University, Jeonju, Korea; University Hospital of Münster, GERMANY

## Abstract

Human sperm have to undergo a maturational process called capacitation in the female reproductive tract. Capacitation confers upon the sperm an ability to gain hypermotility and undergo acrosome reaction. Previous studies have suggested that seminal plasma proteins induce the capacitation of sperm in the female reproductive tract for the successful fertilization of the oocyte. However, the function of seminal plasma proteins in capacitation remains largely unclear. To the end, we found that soluble CD38 (sCD38) in seminal plasma increases the capacitation of sperm via specific interactions between sCD38 and the CD31 on the sperm. Upon the association of sCD38 with CD31, tyrosine kinase Src phosphorylates CD31, a process blocked by Src inhibitors. Shc, SHP-2, Grb2, and SOS, as well as Src kinase were found to associate with the phosphorylated CD31. The sCD38-induced phosphorylation of CD31 initiates a cascade reaction through the phosphorylation of Erk1/2, which results in the acrosome reaction, and sperm hypermotility. These processes were prevented by Src, Ras and MEK inhibitors. Taken together, these data indicate that the sCD38 present in seminal plasma plays a critical role in the capacitation of sperm.

## Introduction

Mammalian seminal plasma is a physiological secretion that originates from multiple glands in the male reproductive tract that plays an important role in the final maturation of the spermatozoa [1]. Capacitation confers upon the sperm an ability to gain hypermotility and undergo acrosomal reaction [2]. The intracellular signaling pathways implicated in capacitation that have been reported include an increase in membrane fluidity, cholesterol efflux, an increase in intracellular Ca^2+^ concentrations, and increased protein tyrosine phosphorylation [3]. Protein tyrosine phosphorylation is an essential aspect of capacitation [4]. Although it has been proposed that seminal plasma proteins, present in secretions from seminal vesicles and prostate glands, regulate the capacitation of sperm [5], the molecular mechanisms and signal transduction pathways involved in this process are not clearly understood.

CD38 is a type II transmembrane glycoprotein with a long C-terminal extracellular domain and a short N-terminal cytoplasmic tail [6]. The extracellular domain of CD38 has bifunctional enzyme activities that catalyze the synthesis of cyclic ADP ribose (cADPR), a calcium second messenger, from nicotinamide adenine dinucleotide (NAD) and the hydrolysis of cADPR to ADPR [7, 8]. Our previous data showed that seminal fluid contains the 37 kDa soluble form of CD38 (sCD38), and the intact form of CD38 was present in prostasomes [9, 10]. Intact CD38 transferred from prostasomes to sperm plays a critical role in progesterone-induced long-lasting Ca^2+^ signaling, which is required for sperm hypermotility [9]. In addition, seminal sCD38 induces the differentiation of tolerogenic dendritic cells in the female uterus, thereby contributing to successful pregnancy by supporting fetomaternal tolerance [10].

CD31, also called platelet endothelial cell adhesion molecule (PECAM)-1, is a 130-kDa type I transmembrane glycoprotein that is expressed in endothelial cells, monocytes, granulocytes, platelets, and sperm [11, 12]. Tyrosine phosphorylation of the CD31 cytoplasmic domain occurs in response to the cross-linking of CD31 itself, or exposure to wheat germ agglutinin (WGA) or CD38 [13–15]. Tyrosine phosphorylation of CD31 has been detected in sperm exposed to WGA, which plays an important role in sperm capacitation [12]. This suggests that some molecules in seminal plasma may play an important role in the regulation of sperm capacitation via CD31. However, the physiological stimulants still remain unknown. In the present study, we showed that sCD38 is present in sufficient quantity in seminal plasma to induce tyrosine phosphorylation of the CD31 in cells and demonstrate that sCD38 has a positive effect on sperm capacitation, which plays an important role in fertility.

## Materials and Methods

### Preparation of Human Specimen

Semen samples were obtained from normal healthy volunteers by masturbation. The study was approved by the Ethics Committee of Human Research at the Chonbuk National University Hospital (IRB#: 2011-01-013) and Informed consent was obtained in written form from each donor. For isolation of sperm by the immediate wash out method, un-liquefied semen was directly added to a 20-fold volume of Bigger, Whitten, and Whittingham (BWW) medium (10 mM HEPES, 20 mM sodium lactate, 5 mM glucose, 0.25 mM sodium pyruvate, 95 mM NaCl, 4.8 mM KCl, 1.3 mM CaCl_2_, 1.2 mM KH_2_PO_4_, and 1.2 mM MgSO_4_ in 25 mM NaHCO_3_ buffer, pH 7.4) [16] and mixed by inverting and then centrifuged at 500 X g for 20 minutes at room temperature. The precipitated sperm was suspended using the same buffer and the wash out procedure was repeated twice. The number of isolated sperm was counted using a Makler chamber (Sefi Medical Instruments, Haifa, Israel) and adjusted to a concentration of 1 to 3 × 10^7^ cells/ml in a suitable medium for each experiment. We performed to determine semen volume, sperm concentration, motility, morphology, and round cells according to 5th edition of the World Health Organization (WHO) manual (2010) for each sample. We only used ejaculates that exhibited normal semen quality that satisfied the following criteria: volume ≥3.0 mL, sperm concentration ≥60 × 10^6^/mL, and progressive (PR) motility ≥50%.

### Purification of recombinant human sCD38

Human sCD38 (a gift from H.C. Lee) was obtained as previously reported with some modification [17]. The *sCD38* transformed *P*. *pastoris* strains were grown at 28°C for 24 hours in 400 mL of buffered glycerol-complex medium (BMGY) and induced with methanol. When the activity of NGD or ε-NAD was maximal, culture media were harvested by centrifugation at 8,000 X g for 20 minutes. The sCD38 in the media was precipitated by 70% ammonium sulfate fractionation at 4°C and the precipitate collected by centrifugation at 14,000 X g for 20 minutes at 4°C. The precipitate was resuspended and dialyzed with 15 mM Tris-HCl pH 7.4. The sample was loaded onto Reactive RED 120-agarose (Sigma-Aldrich), and sCD38 was separated using a linear salt gradient. Fractions showing ADP-ribosyl cyclase activity were pooled, applied to ceramic hydroxyapatite (Bio-Rad), and eluted using a linear phosphate gradient. Thereafter, purified sCD38 was separated with HiLoad 26/600 Superdex 75 (GE Healthcare). Purified sCD38 was loaded onto a High Capacity Endotoxin Removal Spin Column (Pierce) and eluted samples were aliquoted and stored at –70°C until use.

### Imaging of sperm with confocal microscopy

For localization of the interaction between the indicated molecules, we performed immunofluorescence staining as described previously [18http://www.ncbi.nlm.nih.gov/pubmed/19567915] with a slight modification. Briefly, sperm were incubated with sCD38 at 37°C for 30 minutes. The samples cross-linked by 0.5 mM disuccinimidylsuberate (DSS) were transferred into 3.7% paraformaldehyde-PBS and gently mixed. They were kept at 4°C for 1 hour and then washed three times with ice-cold PBS. Sperm were mounted as a smear on glass and air dried and then permeabilized with 0.1% Triton X-100, 1% BSA in PBS at 4°C for 30 min. *In situ* proximity ligation assay (PLA) (Duolink II secondary antibodies and detection kit; Olink Bioscience, Uppsala, Sweden) with the mouse CD38-antibody (Santa Cruz Biotechnology, clone AT1; 1:50) and the rabbit CD31 antibody (Epitomics, clone EP3095; 1:100) was used to detect interaction between sCD38 and CD31. With this method, staining occurred only when the sCD38 was bound to CD31 of sperm. Samples were incubated overnight at 4°C with the two antibodies. The Duolink II Fluorescence Detection Kit with PLA plus and minus probes for mouse and rabbit (Olink Bioscience) was used to visualize the bound antibody pairs, according to the manufacturer’s description. Specimens were mounted with the Duolink Brightfield Mounting Medium (Olink Bioscience).

### Sperm tyrosine phosphorylation and co-immunoprecipitaiton

After isolation of un-capacitated sperm by the immediate wash out method, sperm were incubated with sCD38. Sperm were pelleted by centrifugation at 500 g for 5 minutes and solubilized by incubation in lysis buffer containing 50 mM Tris-HCl, 1 mM EDTA, 150 mM NaCl, 25 mM NaF, 1 mM Na_3_VO_4_, protease inhibitor cocktail, and 1% SDS for 10 minutes at 100°C. Equivalent amounts of protein were separated by SDS-PAGE and transferred to PVDF membranes. Immunodetection of phosphotyrosine residues was performed at 4°C. For co-immunoprecipitation, sCD38-induced sperm were pelleted as above procedure and solubilized using lysis buffer containing PBS, 1 mM MaCl_2_, 25 mM NaF, 1 mM Na_3_VO_4_, protease inhibitor, and 1% Triton X-100. Supernatants were obtained after centrifugation at 15,000 X g for 10 minutes. For immunoprecipitation, cell lysates (800 X g) precleared with protein G-agarose were incubated with anti-CD31 mAb (Cellsignaling, 89C2 clone) or mouse IgG overnight at 4°C and then further incubated with protein G-agarose at 4°C for 1 hour. The immunoprecipitates were washed four times with cell lysis buffer and boiled for 10 minutes. The immunoprecipitated proteins were subjected to western blotting as above procedure and blots were incubated in blocking buffer containing 5% BSA for 2 hours at room temperature and then with primary antibodies (phosphotyrosine [anti-pY], CD38, CD31, Src, Grb2, SHP2, SHC-1, and actin) in blocking buffer overnight at 4°C. The immunoreactive proteins with the respective secondary antibodies were determined using an enhanced chemiluminescence kit (GE healthcare) and exposed to an LAS-1000 ImageReader Lite (Fujifilm, Japan).

### Acrosome reaction

2 × 10^6^ spermatozoa were aliquoted into 1.5 ml tubes. Washed cells were incubated in BWW buffer with or without sCD38 (90 minutes, 37°C). The inhibitors were added for the last 10 minutes of incubation, and then A23187 (10 μM final) was added for 30 minutes. After A23187 treatment, an aliquot of the sperm was spread on slides and allow to air dry. Sperm were permeabilized with cooled methanol for 5 minutes and stained with FITC-*P*. *sativum* agglutinin (PSA) (60 μg/ml) for 1 hour. Slides were viewed using a Nikon TE2000 microscope and 200 sperm were analyzed per slide for the presence (blue staining) or absence (no staining) of the sperm acrosome.

### Computer analysis of sperm motility

The sperm motility analysis was done in accordance with the ESHRE guidelines for the application of computer-assisted sperm analysis (CASA) technology [19]. Prepared sperm were incubated in BWW medium with or without sCD38 (90 minutes, 37°C, 5% CO_2_). The analysis of sperm kinetic parameters was performed using CASA (IVOS; Hamilton Thorne Biosciences, MA). Suspension of sperm was loaded in a flat 20 μm deep disposable sperm analysis chamber (2X-CEL, Hamilton Thorne Biosciences). 1 second tracks were captured at 60 Hz under ×4 dark-field illumination. Instruments were set at: temperature: 37°C; minimum cell size: 3 pixels; video frequency: 60; VAP cutoff: 5.0 μm/s; VSL cutoff: 11.0 μm/s. Hyperactivated sperm were defined as the velocity which is VCL ≥ 150 μm/s, ALH ≥ 7.0 μm/s, LIN ≤ 50%.

### Statistics

**Fig 1:**
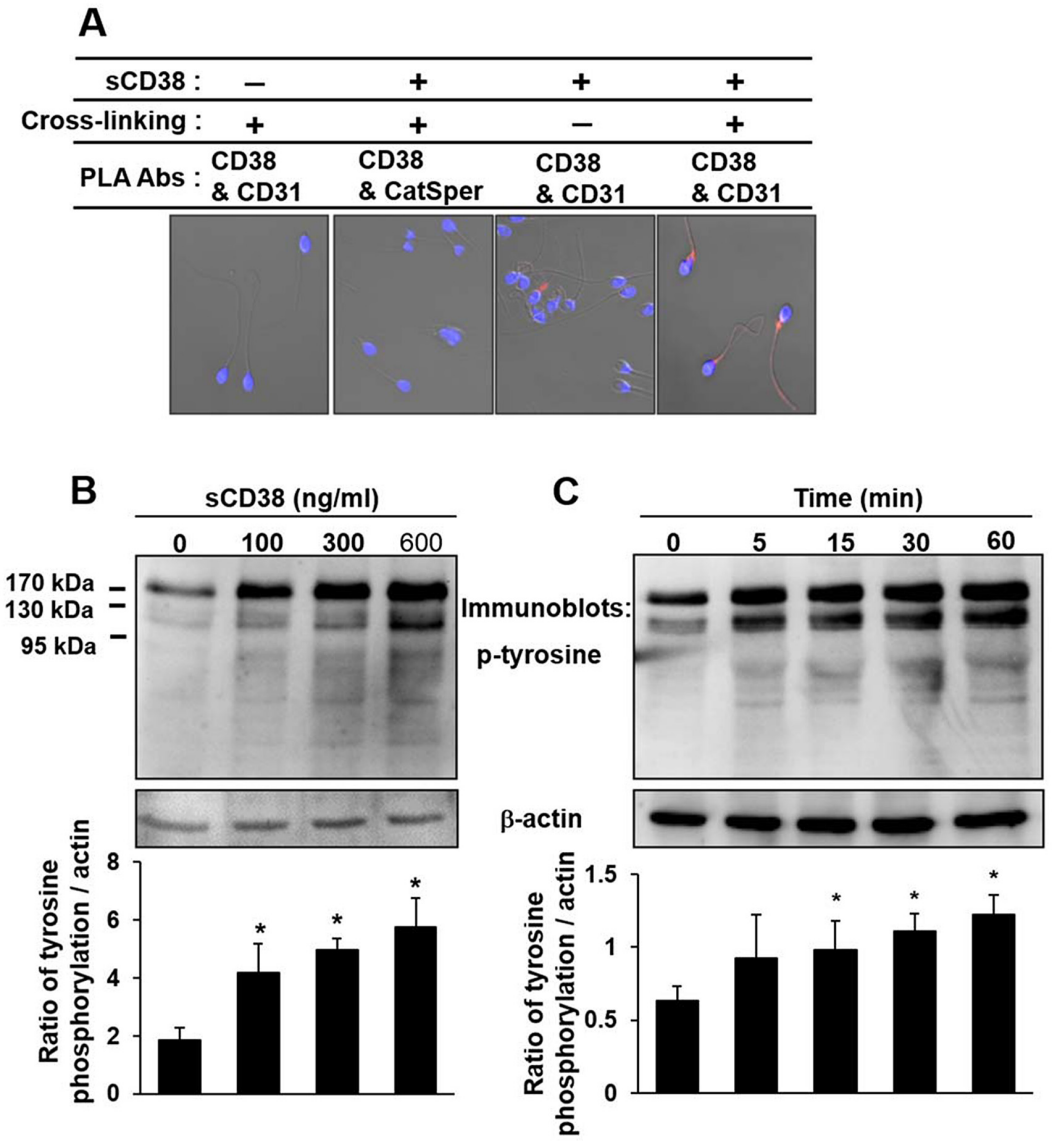
Interaction of sCD38 and CD31 and sCD38-induced tyrosine phosphorylation of proteins in sperm. (A) Interaction of sCD38 and CD31 as demonstrated by PLA on sperm cross-linking with DSS. Human sperm were incubated with or without 2 μg/ml sCD38 for 1 hour at 4°C and cross-linked with 3 mM DSS prepared in DMSO for 30 minutes at room temperature, then fixed with formalin. Sperm were subjected to immunofluorescence staining with antibodies against CD38, CD31 or CatSper, and then visualized with confocal laser-scanning microscopy. Isolated human sperm by the immediate washout were incubated in BWW medium supplemented with different concentration of sCD38 (B) and were stimulated with 600 ng/ml sCD38 for the indicated times (C). Western blot analyses were performed with anti-pY antibodies or anti-actin as controls.

**Fig 2:**
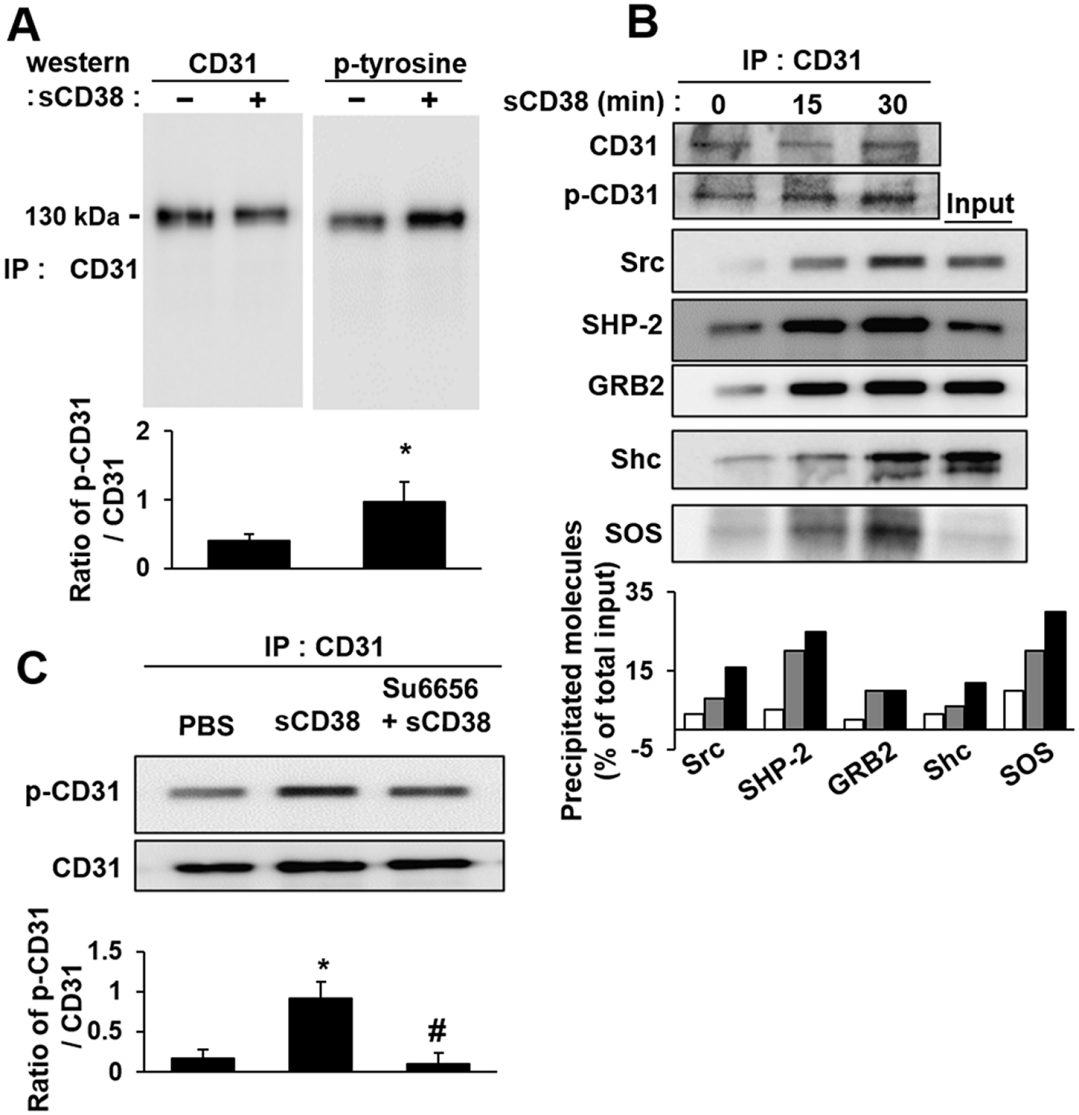
The heterophilic activation of CD31 by sCD38 transduces outside-in signal. (A) Human sperm were stimulated with sCD38 for 10 minutes. The sperm lysates were immunoprecipitated with anti-CD31 antibodies and then immunoblotted with anti-pY antibodies. (B) Isolated sperm were stimulated with 600 ng/ml sCD38 for 0 minute, 15 minutes and 30 minutes, and CD31-associated proteins were examined by co-immunoprecipitation. Heterophilic interaction of CD31 by sCD38 enhanced its association with the intracellular protein Src, SHP2 and Grb2, which were detected by anti-Src, GRB2, Shc and SOS antibodies. (C) Src is required for CD31 tyrosine phosphorylation in sperm. Sperm were pre-incubated with 50 μM Su6656. After incubating for 30 minutes at 37°C, CD31 activation was induced by addition of 600 ng/mL sCD38. After a further incubation for 30 minutes, cells were lysed in Triton X-100, and CD31 immunoprecipitates were prepared. Immunoblot analysis was performed to determine the extent of CD31 tyrosine phosphorylation, using an anti-pY antibodies. Input represents ten percent of the content in the cell lysate of the molecules that were immunoprecipitated; in the bottom panel, analyses of band intensity are presented as the percentage of immunoprecipitated proteins per total input.

**Fig 3:**
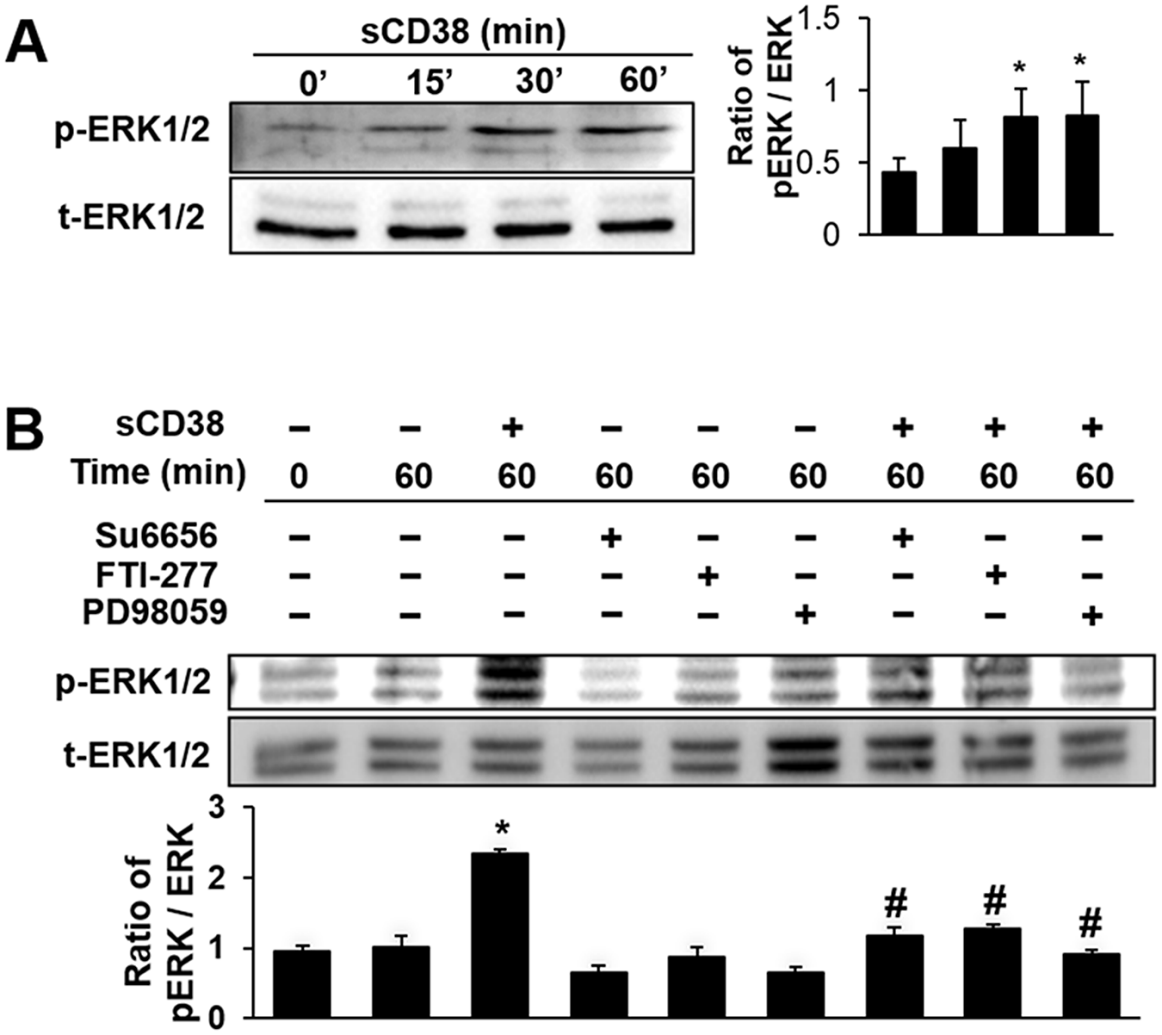
The sCD38-induced tyrosine phosphorylation of sperm protein is mediated by ERK pathway. (A) CD31 activation of human sperm by sCD38 stimulated tyrosine phosphorylation of sperm protein mediated by ERK1/2. Human sperm were incubated in capacitating medium supplemented with sCD38 as time-dependent manner. Following incubation, sperm were solubilized in lysis buffer and prepared for immunoblotting with anti-phospho-ERK1/2 and anti-phospho-p38. Total ERK and p38 was detected as a control for sample loading. (B) Sperm were preincubated with inhibitors (50 μM SU6656, 3 μM FTI-277 and 50 μM PD98059) for 30 min, then treated with (+) or without (-) 600 ng/ml sCD38 for 0 or 60 minutes, sperm proteins were immunoblotted with the anti-phospho-ERK1/2 antibody and total ERK1/2.

**Fig 4:**
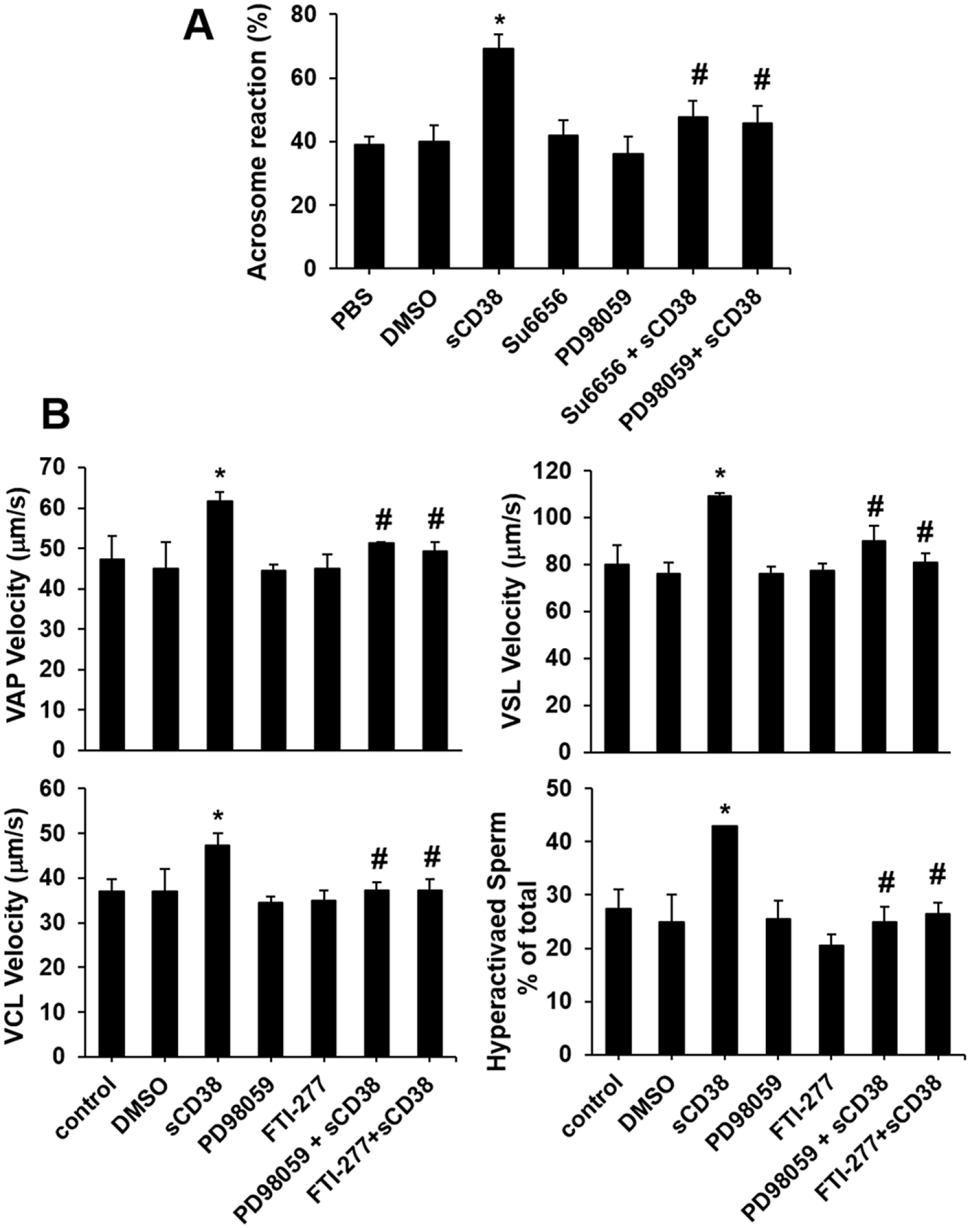
Acrosome reaction and motility of human sperm are increased by sCD38. (A) Human sperm were preincubated in either the presence or absence of sCD38 for 90 minutes. The specific inhibitors were added for the last 10 minutes of the preincubation, and A23187 was added for 30 minutes. The percentage of acrosome reacted cells were determined using FITC-conjugated PSA. *P < 0.05 versus control (PBS treatment); #P < 0.05 versus group treated with sCD38. (B) sCD38-induced sperm motility or hyperactivation was analyzed with the CASA system. Sperm were preincubated in either the presence or absence of the specific inhibitors for 10 minutes and sCD38 (600 ng/ml) was added. Sperm motility was measured using CASA. Results are mean ± S.D. from four experiments. *P < 0.05 versus control (no treatment); #P < 0.05 versus group treated with sCD38.

Results were compared by using analysis of variance. All data were analyzed using SigmaPlot 10.0 software (Systat Software). To evaluate the effects of sCD38 on the various parameters of sperm tyrosine phosphorylation, CD31 tyrosine phosphorylation, Erk, or p38, the t-test was used. The effects of inhibitors on CD31 phosphorylation or Erk phosphorylation and effects sCD38 on parameters, including acrosome reaction or sperm motility, were analyzed using one-way ANOVA. Data represent the mean ± SD in the legends of Figs 1, 2A, 2B and 3A, and data represent the mean ± SEM” in the legends of Figs 3B and 4.

## Results

Seminal fluid contains two forms of CD38, each with molecular weights of 37 kDa and 45 kDa [9, 10]. Also, An in-gel activity assay of CD38 showed two different molecular weight fluorescent bands (S1 Fig); the lower molecular weight form is soluble CD38 (sCD38) that originated from seminal vesicles, and the 45 kDa CD38 form is localized in the prostasomes as an intact form. The findings that plentiful amounts of sCD38 are present in human seminal plasma (0.5 μg/ml∼10.6 μg/ml) [10], and that human sperm express CD31 [12] suggest that the sCD38 in seminal plasma may interact with its target protein, CD31. To examine whether sCD38 interacts with sperm CD31, we performed an *in situ* PLA [18] that enables the detection and quantification of protein–protein interactions in native cells by using both anti-CD38 and anti-CD31 antibodies. Consistent with the previous finding that sCD38 has a low affinity binding domain for a CD31 (14), PLA fluorescence intensity for sCD38-CD31 interaction was only detected with cross-linking, compared to that without cross-linking (Fig 1A). We also performed the in situ PLA experiments using CatSper antibody as a negative control and found no significant signal of association between sCD38 and CatSper. These results indicate that sCD38 interacts with CD31 on the sperm.

Although Nixon et al [12] reported that WGA promotes protein tyrosine phosphorylation in human sperm through CD31, the physiological activation mechanism of CD31 has not been explored. Therefore, we examined the possibility that sCD38 enhances tyrosine phosphorylation of sperm protein through CD31 activation. Indeed, sCD38 induced tyrosine phosphorylation of proteins in a dose- and time-dependent manner (Fig 1B and 1C). Interestingly, sub-physiological concentrations (sub-microgram/ml levels) of sCD38 present in seminal plasma were found to be enough to stimulate the tyrosine phosphorylation of proteins in sperm.

Ligation using specific antibodies against CD31 promotes tyrosine phosphorylation of CD31 within its intrinsic ITIM (immunoreceptor tyrosine-based inhibition motif) domains, resulting in the initiation of CD31 signaling [13]. Therefore, we then examined whether sCD38 is also capable of inducing the tyrosine phosphorylation of CD31. As shown in Fig 2A, an increase in tyrosine phosphorylation of CD31 was observed in sperm stimulated with sCD38 when compared with the non-stimulated control sperm. Since tyrosine kinase Src has shown to be a strong candidate for mediating the tyrosine phosphorylation of CD31 [20], we examined whether the binding of sCD38 to CD31 results in the recruitment of tyrosine kinase Src, along with other intracellular signaling molecules. This notion was confirmed by the finding that sCD38 induced the phosphorylation of CD31 in a time-dependent manner (Fig 2B). Moreover, the tyrosine phosphorylation of CD31 correlated with its increased association with other proteins, including Src, Shc, SHP-2, GRB2, and SOS, which are involved in the activation of the MAPK/ERK-signaling pathways in other cells [21]. Consistent with the above findings, the Src tyrosine kinase inhibitor Su6656 inhibited the sCD38-induced CD31 phosphorylation (Fig 2C).

Because CD31-associated molecules are involved in the activation of MAPK/ERK-signaling pathways [21], we examined whether sCD38 induces the activation of MAPK/ERKs in sperm. Western blot analysis showed that sCD38 treatment activated MAPK/ERKs in a time-dependent manner, but not p38 (S1 Fig). To delineate further down-stream in the CD31 to ERK pathway, we tested the effects of inhibitors of Src, Ras, and MEK on sCD38-induced phosphorylation of Erk1/2 in sperm. All inhibitors of Src, Ras, and MEK blocked sCD38-induced phosphorylation of Erk1/2 (Fig 3B), suggesting that ERK is activated by molecules downstream to CD31. Evidence for the involvement of ERK1/2 in the acrosomal reaction was reported in lysophosphatidylcholine-induced human sperm [22]. The MAPK pathway was shown to regulate sperm capacitation and protein tyrosine phosphorylation through Grb2, Ras, Raf, and MEK. Furthermore, evidence regarding the role of ERK in sperm motility has been reported in fowl sperm [23]. Likewise, a study reported that ERK1/2 stimulates forward motility and hyperactivated motility, and are involved in the acrosomal reaction in the sperm, which is induced by PMA [24]. Therefore, we determined if sCD38 is required for the acrosomal reaction and sperm motility. The addition of sCD38 as a capacitation inducer for sperm significantly increased A23187-induced acrosomal reaction, which was inhibited by Su6656 or PD98059 (Fig 4A). Treatment with sCD38 displayed increased sperm motility in the CASA [as assessed by average path velocity (VAP), straight-line velocity (VSL), and curvilinear velocity (VCL)] (Fig 4B). The increased sperm motility elicited by sCD38 was significantly inhibited by PD98059 or FTI-277. Sperm hyperactivation is important to fertilization, and is characterized by high-amplitude asymmetrical flagellar bending [25]. Therefore we analyzed sperm hyperactivation by CASA, and found that sCD38 induced the hyperactivation of sperm, which was inhibited by pretreatment with PD98059 or FTI-277.

## Discussion

CD31 is a member of the immunoglobulin (Ig) superfamily, and is composed of 6 extracellular Ig domains, a transmembrane region, and a cytoplasmic tail [13]. It is generally considered that CD31 functions as both a hemophilic and heterophilic adhesion molecule, which facilitates communication between cells [11, 13]. CD31 mediates inhibitory signals in immune cells [26, 27]. Recently, it was reported that CD31 receptor globulin-treated mice showed less neovascularization and intraplaque hemorrhaging when compared with the control in the atherosclerosis mouse model [28]. Consequently, CD31 plays a key role in the prevention and treatment of diseases caused by a pathogenic immune response. On the other hand, CD31’s involvement in receptor-mediated signal pathways regulates the tyrosine phosphorylation of sperm protein, which is involved in the capacitation of human sperm [12]. However, the physiological molecule that is involved in this process was not identified in seminal plasma or the female reproductive tract.

Mammalian semen has been reported to possess a NAD-glycohydrolase enzyme [29]. We recently showed that intact CD38 is present in prostasomes, exocytic cell vesicles derived from prostate glands, which are fused to the midpiece of the sperm in a pH-dependent manner, regulating the progesterone-stimulated Ca^2+^ signaling of sperm [9]. Furthermore, we identified an additional, soluble form of sCD38 in seminal plasma other than the intact CD38 in prostasomes [10]. sCD38 was initially found in normal and pathological fluids, and its binding to human myeloid cells is mediated by CD31 [30]. However, its physiologic function has not yet been elucidated. Interestingly, sCD38 is present in the μg/ml range in seminal plasma. Therefore, sCD38 in seminal plasma is present in sufficient concentrations for the regulation of target cells in the female reproductive tract.

Our data demonstrate that sCD38 present in seminal plasma induces sperm capacitation through CD31. In human sperm, we found that purified sCD38 specifically interacted with CD31, resulting in the phosphorylation of CD31 and the tyrosine phosphorylation of sperm protein (Figs 1 and 2A). A previous study reported that the signaling pathway of Shc, Grb2, Ras, and Erk1/2 was involved in the capacitation of human sperm [22]. Our data also showed that CD31 activated by sCD38 recruited Shc-Grb2-SOS1, which formed a signaling-complex to regulate ERK-signaling pathways (Fig 2B and 2C). Src regulates the phosphorylation of CD31 (Fig 2B), which creates sites for the binding of SHP-2, which in turn provides docking sites for signaling-complexes such as Shc, Grb2, and SOS1 [21]. The inhibition of Src or Ras using specific inhibitors resulted in the inhibition of CD31 activation-mediated Erk1/2 phosphorylation (Fig 3B), suggesting that Src and Ras, which are upstream to Erk1/2, are involved in sCD38-induced CD31 signaling. Most interestingly, sCD38 significantly increased the acrosomal reaction in sperm, which was then inhibited by the inhibition of Src or Erk1/2 (Fig 4A). Sperm motility was improved by sCD38 as well, and inhibitors of Ras or Erk1/2 attenuated the sCD38-induced enhancements to motility (Fig 4B).

Seminal plasma promotes immune tolerance in the female reproductive tract. Although this relationship with regards to pregnancy has been convincingly demonstrated, the molecules involved in this process were not clearly identified in the seminal plasma. In our previous study, we demonstrated that seminal sCD38 confers DCs with immunoregulatory potential, and that these DCs are crucial for fetomateral tolerance [10]. Therefore, sCD38 has a dual function in the female reproductive tract, one which regulates maternal immune tolerance and another that induces sperm capacitation.

## Supporting Information

S1 Fig The p38 pathway is not involved in sCD38-induced tyrosine phosphorylation of sperm protein. The sperm were stimulated with sCD38 in BWW medium, and sperm were solubilized in lysis buffer and prepared for immunoblotting with anti-phospho-p38. Total p38 was detected as a control.
(PDF)
