# Supplementary figures and images for: Seminal CD38 Enhances Human Sperm Capacitation through Its Interaction with CD31
Source: PLoS One. 2015 Sep 25;10(9):e0139110. doi: 10.1371/journal.pone.0139110 (PMC4583300; doi:10.1371/journal.pone.0139110)

S1 Fig

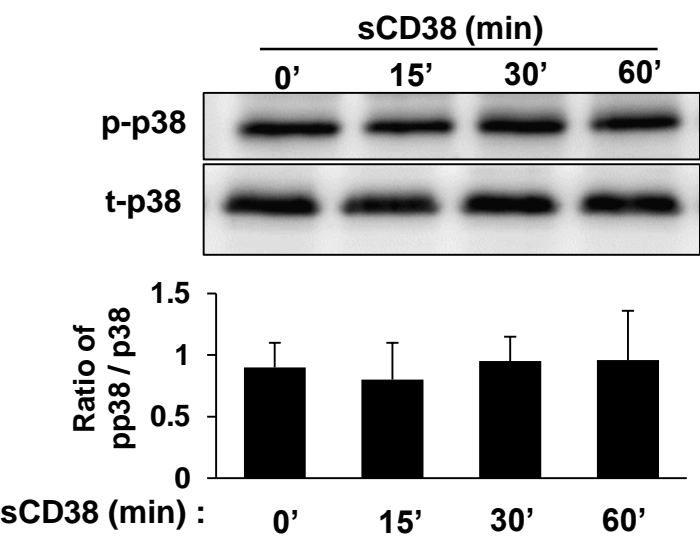

Supplement: S1 Fig — The sperm were stimulated with sCD38 in BWW medium, and sperm were solubilized in lysis buffer and prepared for immunoblotting with anti-phospho-p38. Total p38 was detected as a control. (PDF) [file pone.0139110.s001.pdf]
